# A teleost CD46 is involved in the regulation of complement activation and pathogen infection

**DOI:** 10.1038/s41598-017-15124-y

**Published:** 2017-11-03

**Authors:** Mo-fei Li, Zhi-hai Sui, Li Sun

**Affiliations:** 1 Key Laboratory of Experimental Marine Biology, Institute of Oceanology, Chinese Academy of Sciences, Qingdao, China; 2 Laboratory for Marine Biology and Biotechnology, Qingdao National Laboratory for Marine Science and Technology, Qingdao, China; 3 University of Chinese Academy of Sciences, Beijing, China

## Abstract

In mammals, CD46 is involved in the inactivation of complement by factor I (FI). In teleost, study on the function of CD46 is very limited. In this study, we examined the immunological property of a CD46 molecule (CsCD46) from tongue sole, a teleost species with important economic value. We found that recombinant CsCD46 (rCsCD46) interacted with FI and inhibited complement activation in an FI-dependent manner. rCsCD46 also interacted with bacterial pathogens via a different mechanism to that responsible for the FI interaction, involving different rCsCD46 sites. Cellular study showed that CsCD46 was expressed on peripheral blood leukocytes (PBL) and protected the cells against the killing effect of complement. When the CsCD46 on PBL was blocked by antibody before incubation of the cells with bacterial pathogens, cellular infection was significantly reduced. Consistently, when tongue sole were infected with bacterial pathogens in the presence of rCsCD46, tissue dissemination and survival of the pathogens were significantly inhibited. These results provide the first evidence to indicate that CD46 in teleosts negatively regulates complement activation via FI and protects host cells from complement-induced damage, and that CD46 is required for optimal bacterial infection probably by serving as a receptor for the bacteria.

## Introduction

The complement system is a crucial constituent of the immune system and provides effective protection via mechanisms involving both innate and adaptive immune defenses^1,2^. The complement system consists of three pathways of activation: the classical pathway, the alternative pathway, and the lectin pathway^3^. The three pathways converge at the cleavage of C3 to C3a and C3b, which leads to a series of events involving cleavage of C5 and assembly of C5b, C6, C7, C8, and C9 to generate the membrane attack complex that induces osmotic lysis of the target cells^4,5^.

Complement activation is regulated by many factors^6^. Factor I, a serine protease, is one of the complement regulatory proteins^7^. In humans, factor I regulates the complement activation cascades of both classical and alternative pathways by cleavage and degradation of C4b and C3b in the presence of certain cofactors, thereby preventing the assembly of the C3 and C5 convertases^8–10^. CD46 is a cell surface molecule that functions in monomeric form as a key regulator of the classical and alternative complement activation cascades^11^. CD46 is a cofactor of factor I and regulates complement activation by facilitating the proteolysis of deposited C3b and C4b by factor I, thus preventing amplification of the complement cascade on the cells on which CD46 is expressed^12,13^. CD46 consists of four homologous complement control protein (CCP) repeats, a serine-threonine-proline-rich (STP) domain, a transmembrane hydrophobic domain, a cytoplasmic anchor, and a cytoplasmic tail^14,15^.

In addition to its role in complement activation, CD46 is also employed as a cellular receptor by several viruses and bacteria^11^. Human-specific pathogens targeting CD46 include measles virus, adenovirus groups B and D, herpes virus, *Neisseria gonorrhoeae*, *Neisseria meningitidis*, *Streptococcus pyogenes*, *Escherichia coli*, and *Fusobacterium nucleatum* ^16–19^. In bovine, CD46 is a receptor for bovine viral diarrhea virus, an enveloped RNA virus^20^. A recent study indicated a direct link between CD46 and components of the autophagy machinery^17^. Recognition of pathogens by CD46 is thought to trigger autophagy, which serves as a critical step in controlling infection^11^.

Studies on fish complement system have revealed the existence of almost all the orthologues of mammalian complement components in teleost, and multiple isoforms of some components are present in fish^21^. A few complement molecules, such as C3 and C1q, have been reported to be highly conserved in terms of functional properties between teleosts and mammals, though the immune functions of most fish complement components are unknown^21,22^. CD46-like sequences have been identified in several teleost species; however, only two reports on CD46 have been documented^23,24^. In one of these reports, a recombinant Chinese hamster ovary cell line expressing carp CD46 showed significantly increased tolerance against complement lysis and less deposition of C3, and that anti-CD46 antibody enhanced the depositions of C3 on autologous erythrocytes^23^. Another study examined the expression patterns of membrane-bound regulators of complement activation (gTecrem) in peripheral blood leukocytes (PBL) and erythrocytes of ginbuna crucian carp^24^. Despite these studies, the biological functions of CD46 in fish, especially in host fish *in vivo*, remain to be investigated. The present study, with an aim to gain more insights into the function of teleost CD46, we examined the biological role in complement activation and during bacterial infection in tongue sole (*Cynoglossus semilaevis*).

## Results

### The sequence feature of CsCD46

**Figure 1:**
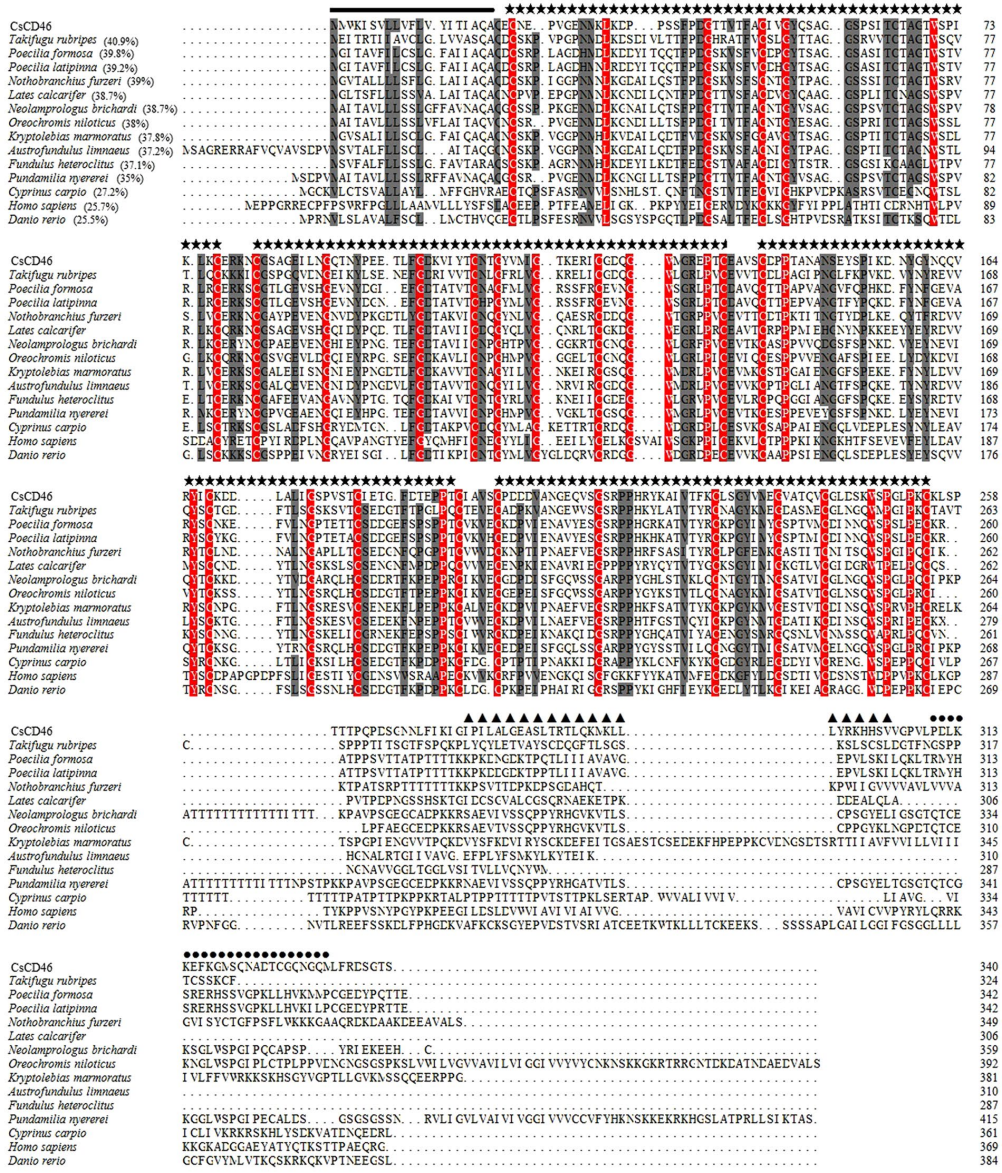
Alignment of the sequences of CsCD46 homologues. Dots denote gaps introduced for maximum matching. Numbers in brackets indicate overall sequence identities between CsCD46 and the compared sequences. The consensus residues are in red, the residues that are ≥75% identical among the aligned sequences are in grey. The signal peptide sequence is indicated by a black line, the CCP domain is indicated by star signal, the transmembrane region is indicated by triangles, and the intracellular region is indicated by circles. The GenBank accession numbers of the aligned sequences are as follows: *Takifugu rubripes*, XP_011616176.1; *Poecilia formosa*, XP_016527734.1; *Poecilia latipinna*, XP_014910555.1; *Nothobranchius furzeri*, XP_015822576.1; *Lates calcarifer*, XP_018539190.1; *Neolamprologus brichardi*, XP_006794358.1; *Oreochromis niloticus*, XP_013130666.1; *Kryptolebias marmoratus*, XP_017274702.1; *Austrofundulus limnaeus*, XP_013866187.1; *Fundulus heteroclitus*, XP_012731019.1; *Pundamilia nyererei*, XP_013770344.1; *Cyprinus carpio*, BAM68364.1; *Homo sapiens*, ABK81636.1; *Danio rerio*, BAM68365.1.

CsCD46 is composed of 340 amino acid residues with a calculated molecular mass of 36.8 kDa and a theoretical pI of 5.74. CsCD46 is a transmembrane protein with a signal peptide sequence (residues 1 to 20), an extracellular region containing four CCP modules (residues 23 to 77, 82 to 135, 140 to 194, and 199 to 254), a transmembrane region (residues 276 to 304), and an intracellular region (residues 310 to 332). CsCD46 was predicted to localize in the cytoplasmic membrane and contained 11 putative O-glycosylation sites. CsCD46 shared 25.5–40.9% overall sequence identities with CD46 from a number of teleost species including *Takifugu rubripes*, *Poecilia formosa*, *Poecilia latipinna*, *Nothobranchius furzeri*, *Lates calcarifer*, *Neolamprologus brichardi*, *Oreochromis niloticus*, *Kryptolebias marmoratus*, *Austrofundulus limnaeus*, *Fundulus heteroclitus*, *Pundamilia nyererei*, *Cyprinus carpio*, and *Danio rerio* (Fig. 1). CsCD46 also shared 25.7% overall sequence identity with human CD46, mainly in the CCP regions (Fig. 1).

### Effect of recombinant CsCD46 (rCsCD46) on complement activation

**Figure 2:**
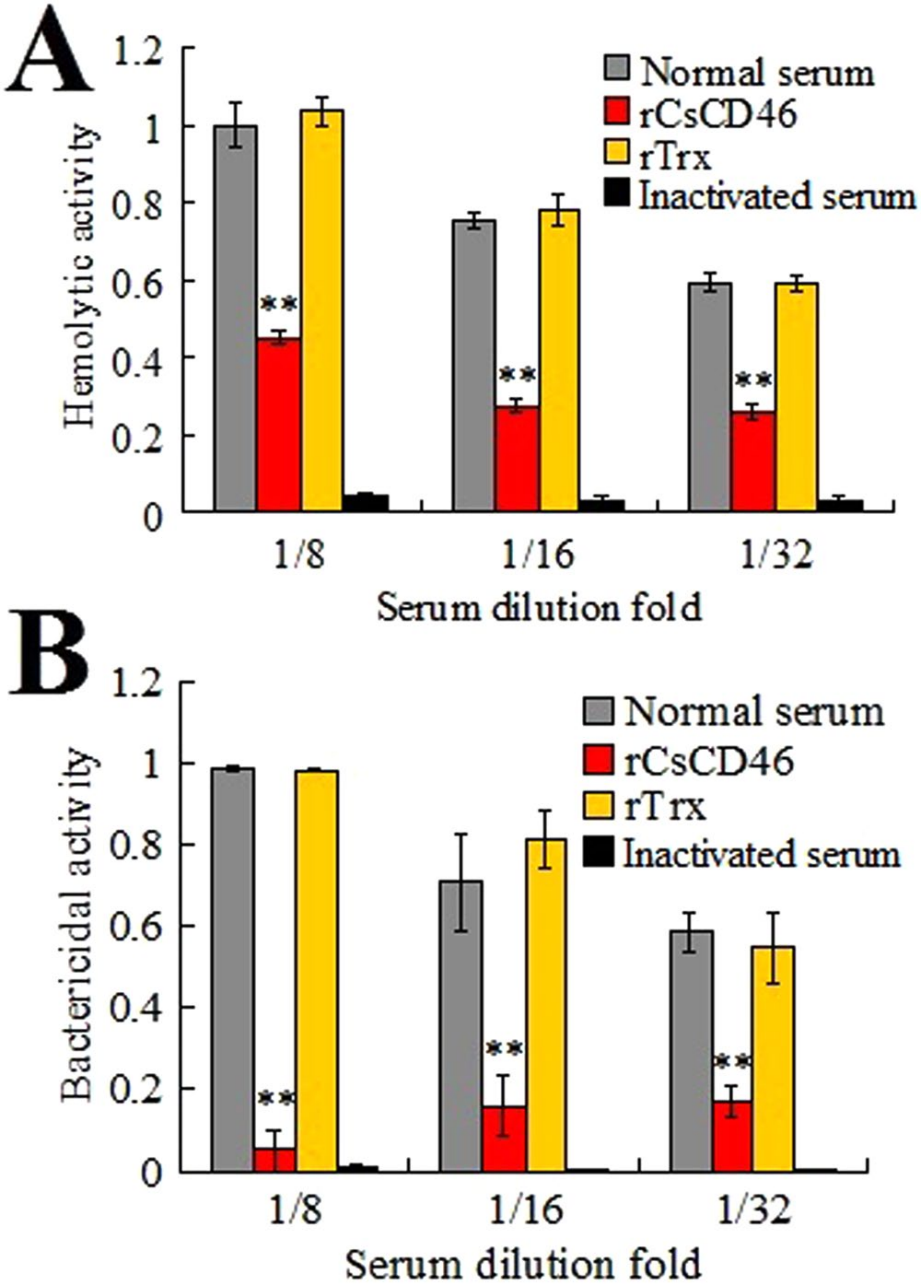
Effect of rCsCD46 on complement activation. Normal and inactivated serum of tongue sole in different dilutions was incubated with or without rCsCD46, rTrx, and the hemolytic (**A**) and bactericidal (**B**) activities of the serum were subsequently determined. Data are the means of three independent experiments and presented as means ± SEM. ^**^ *P* < 0.01.

To examined the potential effect of rCsCD46 on complement activity, dilutions of tongue sole serum were incubated with rCsCD46 or the control protein rTrx that was purified under the same condition as rCsCD46 (Fig. S1). Subsequent analysis showed that serum treated with rCsCD46 at 8-, 16-, and 32-fold dilutions exhibited significantly lower hemolytic and bactericidal activities than untreated normal serum or rTrx-treated serum (Fig. 2), suggesting that rCsCD46 inhibited complement activation.

### Involvement of factor I in rCsCD46-mediated effect on complement

**Figure 3:**
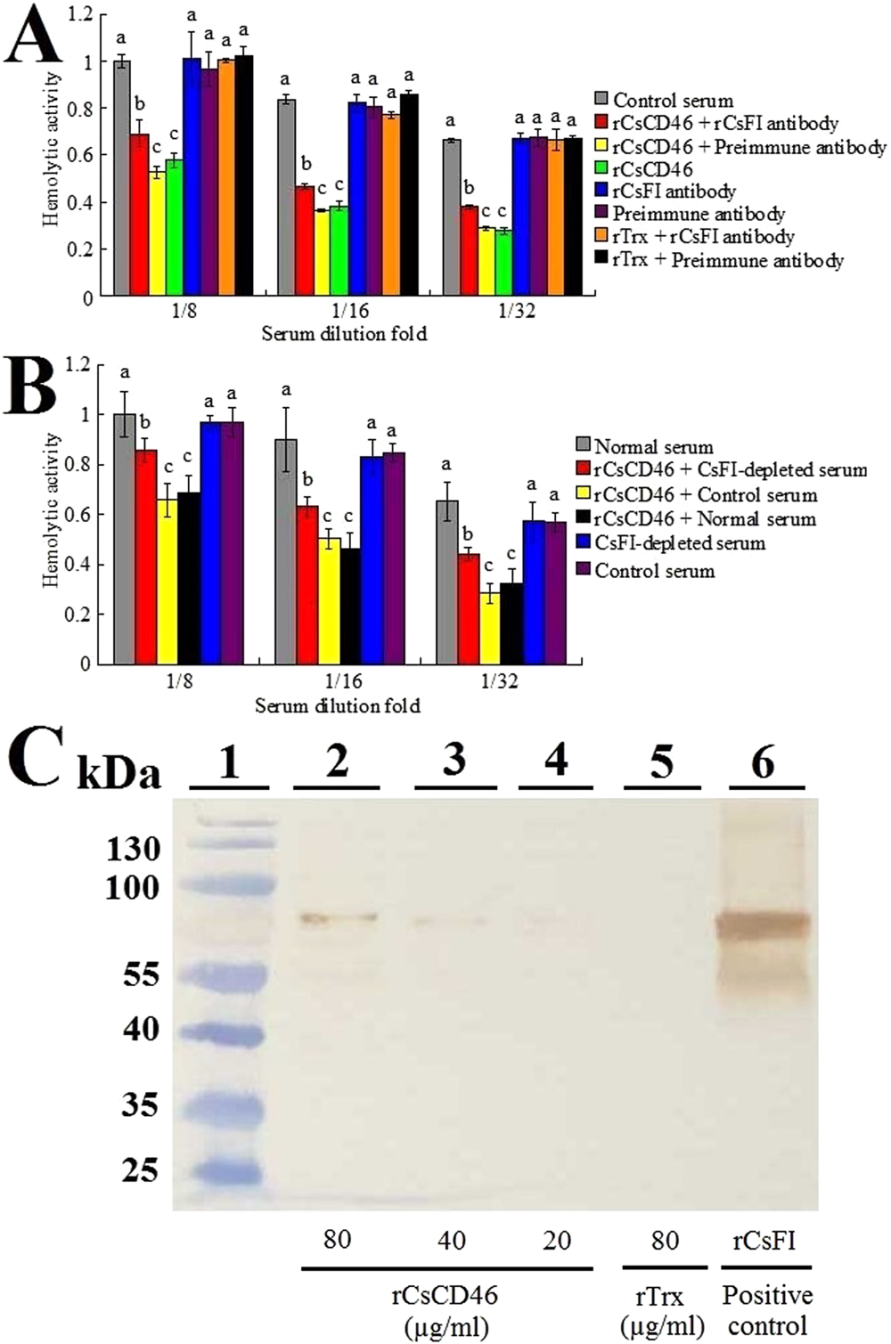
Involvement of CsFI in rCsCD46-mediated effect on complement activation. (**A**) Tongue sole serum in various dilutions was incubated with or without (control) rCsCD46 plus anti-rCsFI antibody, rCsCD46 plus preimmune antibody, rCsCD46, anti-rCsFI antibody, preimmune antibody, rTrx plus anti-rCsFI antibody, and rTrx plus preimmune antibody. The hemolytic activity of the serum was subsequently determined. (**B**) Dilutions of tongue sole serum that had been subjected to anti-rCsFI antibody absorption to deplete CsFI or anti-rTrx antibody absorbance (control serum) were incubated with rCsCD46, and the hemolytic activity of the serum was determined. (**C**) Interaction between rCsCD46 and CsFI. Tongue sole serum treated with different concentrations of rCsCD46 (80 µg/ml, 40 µg/ml, and 20 µg/ml) or Trx (80 µg/ml) was immunoprecipitated with anti-His antibody and then immunoblotted with anti-rCsFI antibody. For (**A**) and (**B**), data are the means of three independent experiments and presented as means ± SEM. Values with different letters indicate significantly different (*P* < 0.05).

With the above observation, we wondered whether factor I was involved in rCsCD46-mediated effect on complement. To investigate this question, tongue sole factor I (CsFI) in the serum was blocked by antibody against rCsFI, and its effect on rCsCD46 activity was subsequently determined. The results showed that for serum treated with rCsCD46 plus anti-rCsFI antibody, the hemolytic activity was significantly lower than that of the control serum, but significantly higher than that of the serum treated with rCsCD46 or rCsCD46 plus preimmune antibody (Fig. 3A), suggesting that the presence of anti-rCsFI antibody significantly reduced the inhibitory effect of rCsCD46 on complement activation. In contrast, treatment with anti-rCsFI antibody and preimmune antibody alone or plus rTrx had no apparent effect on the hemolytic activity. Similar to these results, when rCsCD46 was incubated with serum from which CsFI had been removed by affinity column absorption to anti-rCsFI antibody, the hemolytic activity of the serum was significantly increased compared with rCsCD46-treated normal serum (Fig. 3B). Co-IP and Western blot analyses showed that following incubation with serum, rCsCD46 was co-immunoprecipated with CsFI (Fig. 3C), suggesting that rCsCD46 interacted directly with serum CsFI. However, when rCsCD46 was incubated with rCsFI, no binding between these two proteins was detected (data not shown), suggesting that the interaction between rCsCD46 and rCsFI depended on other factors in the serum.

### Protective effect of CsCD46 on PBL against complement damage

**Figure 4:**
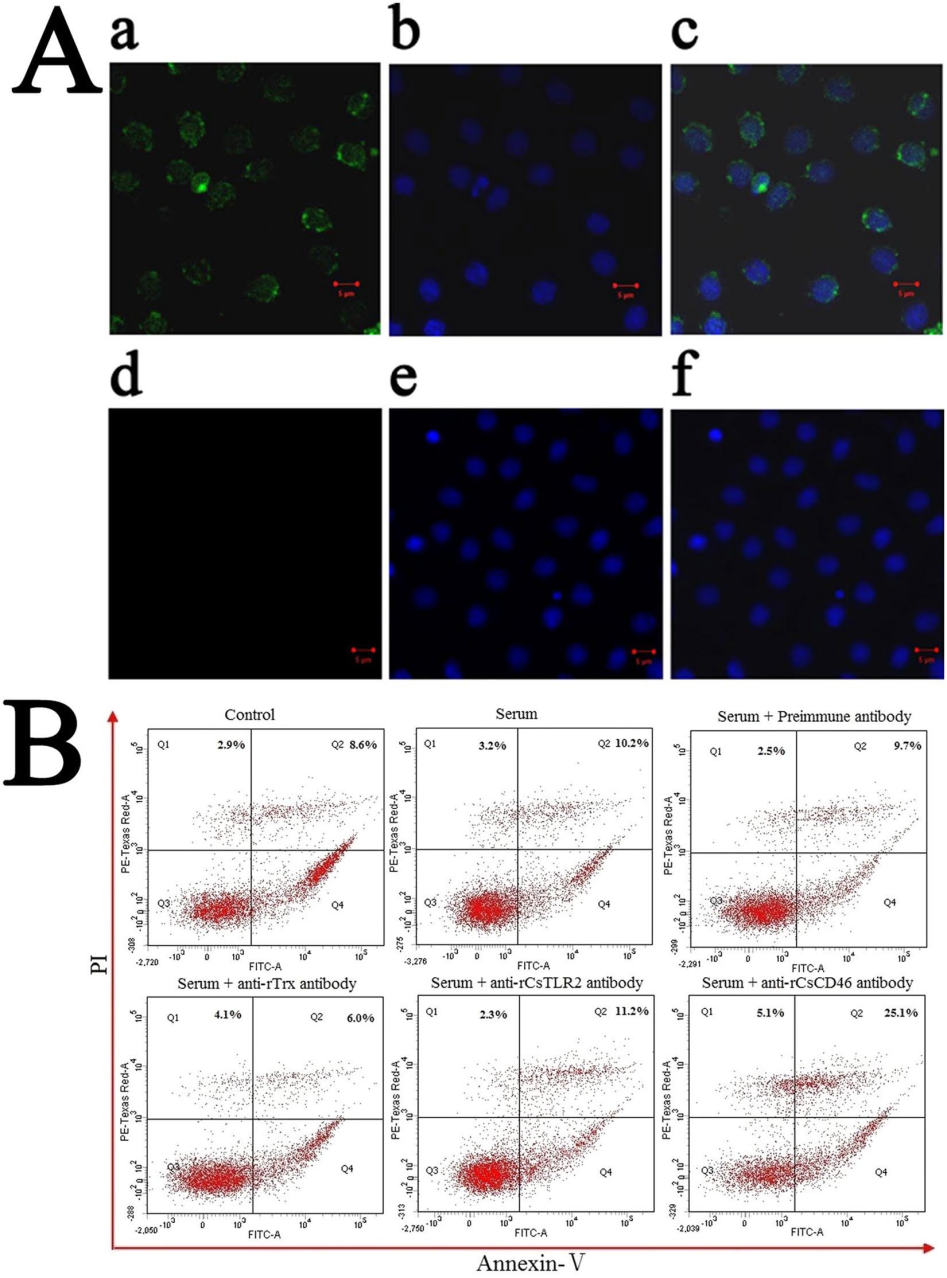
Expression of CsCD46 on peripheral blood leukocytes (PBL) and its effect on complement damage. (**A**) PBL were incubated with anti-rCsCD46 antibody (Aa and Ab) or anti-rTrx antibody (Ad and Ae) and then treated with FITC-labeled secondary antibody and stained with DAPI. The cells were subjected to microscopy with green fluorescence light (Aa and Ad) or blue fluorescence light (Ab and Ae). Ac is a merged image of Aa and Ab; Af is a merged image of Ad and Ae. (**B**) PBL were treated with serum in the presence or absence of anti-rCsCD46 antibody, anti-rTrx antibody, preimmune antibody, or anti-CsTLR2 antibody for 1 h; the control cells were untreated with serum or antibody. Cellular damage was then determined by Annexin V-PI assay.

Immunofluorescence microscopy showed that when PBL was treated with anti-rCsCD46 antibody, the antibody was found to be bound on the cells, whereas no cell-associated antibody was detected in PBL treated with anti-rTrx antibody (Fig. 4A), suggesting that CsCD46 was expressed by and exposed on the surface of PBL. To examine whether the CsCD46 on PBL had any effect against serum-induced cellular damage, PBL were treated with serum in the presence or absence of anti-rCsCD46 antibody, and cell death was determined by flow cytometry. The results showed that in PBL treated with serum plus anti-rCsCD46 antibody, the amount of cell death (30.2% ± 2.8%) was significantly (*P* < 0.01) higher than that in the PBL treated with serum alone (13.4% ± 1.5%) (Fig. 4B). In contrast, the amounts of cell death in PBL treated with serum plus anti-rTrx antibody (10.1% ± 1.1%), anti-rCsTLR2 antibody (13.5% ± 1.9%), and preimmune antibody (12.2% ± 1.3%) were comparable to that in the PBL treated with serum alone (Fig. 4B). When PBL were treated with the serum that had been depleted of factor I by anti-rCsFI antibody, the amount of cell death (31% ± 3.2%) was significantly higher than that of the control cells treated with normal serum (13.4% ± 1.5%) (Fig. S2).

### Binding of rCsCD46 to bacteria via a different mechanism from that for binding to CsFI

**Figure 5:**
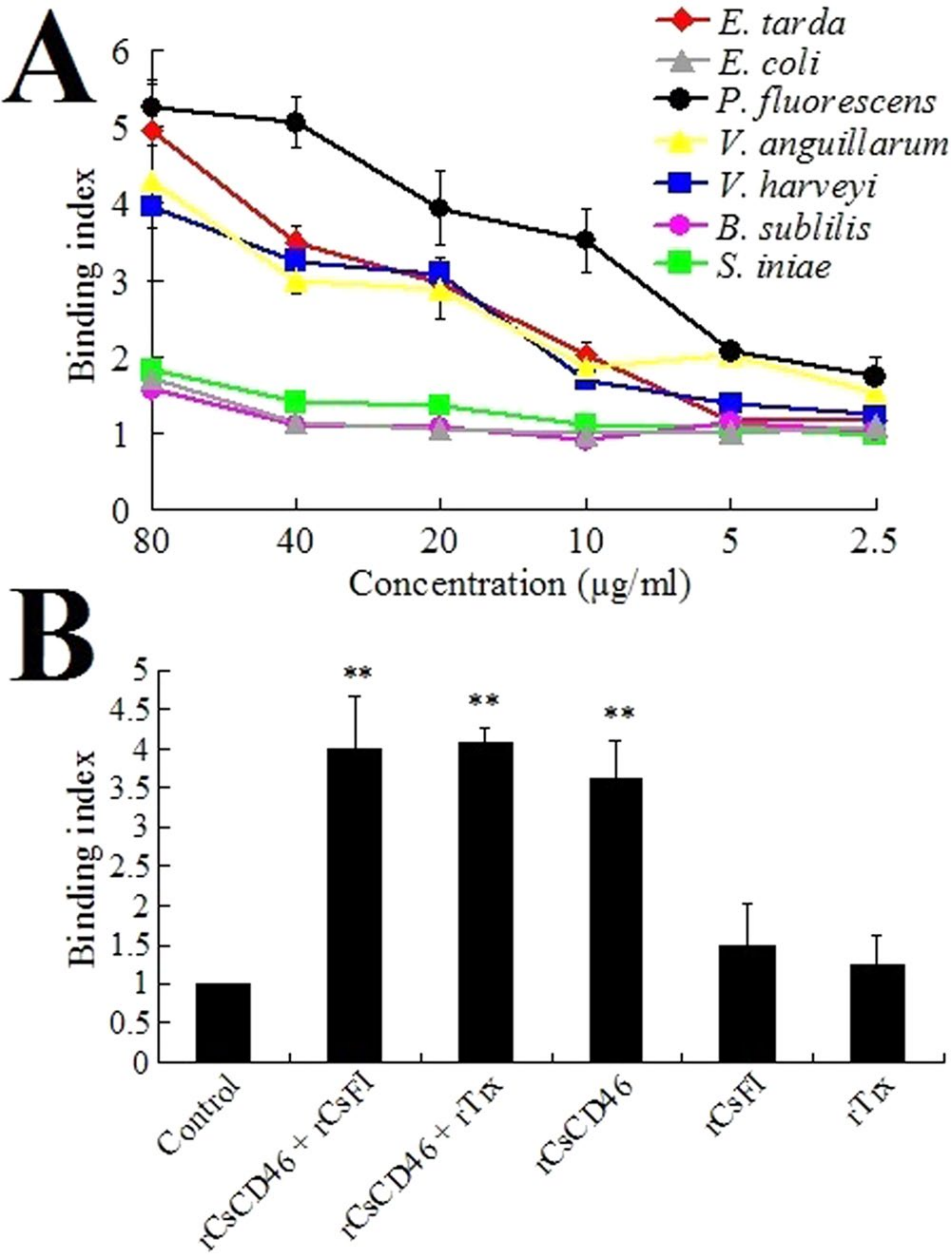
Binding of rCsCD46 to bacteria. (**A**) *Edwardsiella tarda*, *Escherichia coli*, *Pseudomonas fluorescens*, *Vibrio anguillarum*, *Vibrio harveyi*, *Bacillus subtilis*, and *Streptococcus iniae* were incubated with or without (control) different concentrations of rCsCD46, and bacteria-protein binding was determined by ELISA. (**B**) *P. fluorescens* was incubated with or without (control) rCsCD46 plus rCsFI, rCsCD46 plus rTrx, rCsCD46, rCsFI, or rTrx (all proteins were at a final concentration of 40 µg/ml), and protein-bacteria binding was determined as above. Data are the means of three independent assays and presented as means ± SEM. **P < 0.01.

To examine the potential of rCsCD46 to interact with bacteria, rCsCD46 at different concentrations was incubated with the Gram-negative bacteria *Edwardsiella tarda*, *Escherichia coli*, *Pseudomonas fluorescens*, *Vibrio anguillarum*, and *Vibrio harveyi*, and the Gram-positive bacteria *Bacillus subtilis* and *Streptococcus iniae*. Among these bacteria, *E. tarda*, *P. fluorescens*, *V. anguillarum*, *V. harveyi*, and *S. iniae* are common fish pathogens. Subsequent ELISA analysis showed that rCsCD46 exhibited apparent binding to *E. tarda*, *V. harveyi*, *V. anguillarum*, and *P. fluorescens* in a dose-dependent manner (Fig. 5A). Relatively high binding indexes were observed with *P. fluorescens* and *E. tarda*. In contrast, heat-inactivated rCsCD46 or rTrx did not bind any bacteria (data not shown). Plate count analysis showed that rCsCD46 had no apparent effect on the survival of *E. tarda* and *P. fluorescens* (data not shown). To examine whether rCsCD46 bound bacteria and CsFI via the same site in rCsCD46, *P. fluorescens* was incubated with rCsCD46 in the presence or absence of rCsFI. Subsequent ELISA analysis showed that the presence of rCsFI had no significant effect on the binding index of rCsCD46 to bacteria (Fig. 5B).

### Effect of CsCD46 on bacterial infection

#### *In vitro* effect on cellular infection

**Figure 6:**
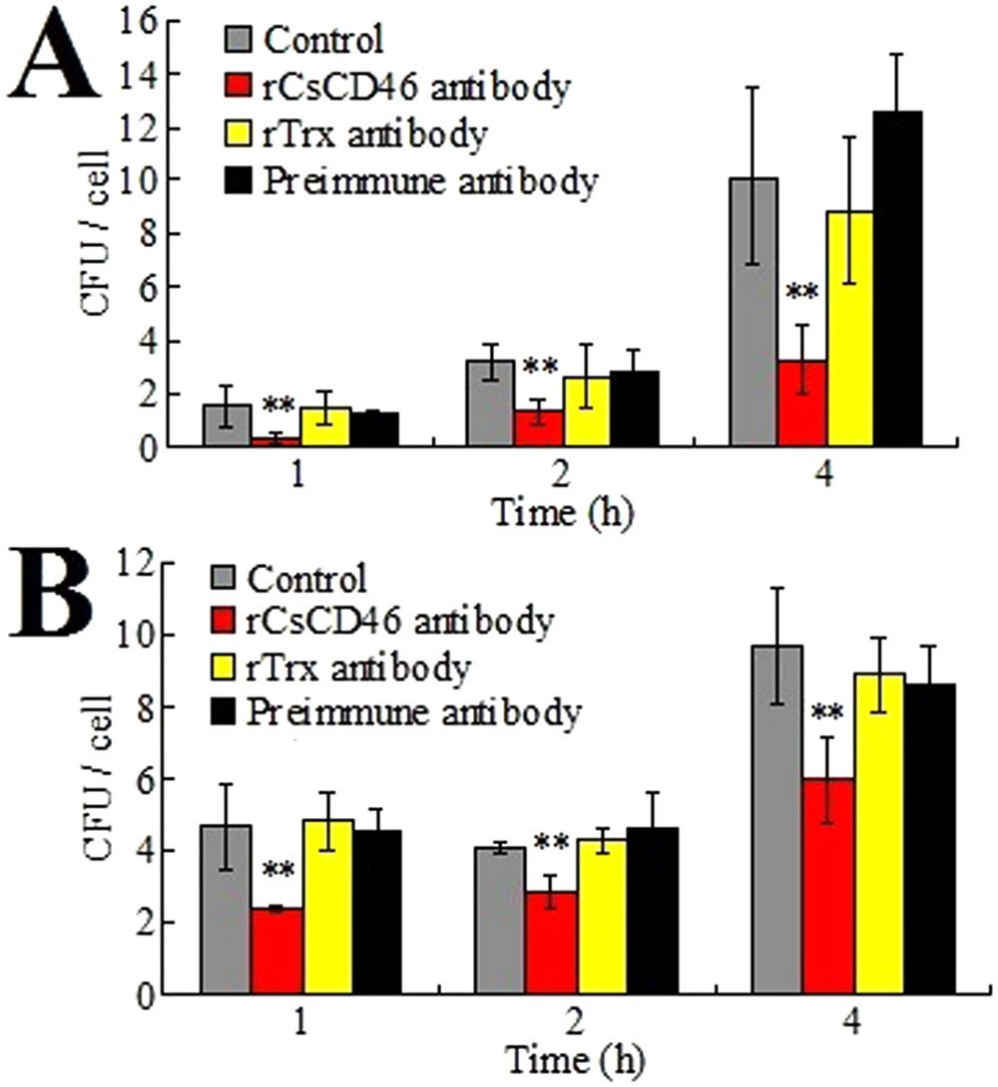
Effect of CsCD46 on bacterial infection in peripheral blood leukocytes (PBL). *Edwardsiella tarda* (**A**) and *Pseudomonas fluorescens* (**B**) were incubated with PBL in the presence or absence (control) of anti-rCsCD46 antibody, anti-rTrx antibody, or preimmune antibody, and bacterial infection was determined by plate count at various hours post-incubation. Data are the means of three independent assays and presented as means ± SEM. **P < 0.01.

With the above results, which showed that rCsCD46 interacted with fish bacterial pathogens and that PBL expressed CsCD46 on the cell surface, we investigated the role of CsCD46 in bacterial infection. To investigate this question, *E. tarda* and *P. fluorescens* were incubated with PBL in the presence or absence of anti-rCsCD46 antibody or anti-rTrx antibody, and bacterial infection was assessed by plate counts to determine the number of PBL-associated live bacteria at different times of infection. The results showed that at 1 h, 2 h, and 4 h post-infection, the numbers of *E. tarda* recovered from Anti-rCsCD46 antibody-treated PBL were significantly lower than that recovered from control cells or cells treated with anti-rTrx antibody or preimmune antibody (Fig. 6A). Similar results were obtained with *P. fluorescens* (Fig. 6B).

#### *In vivo* effect on tissue dissemination and survival

**Figure 7:**
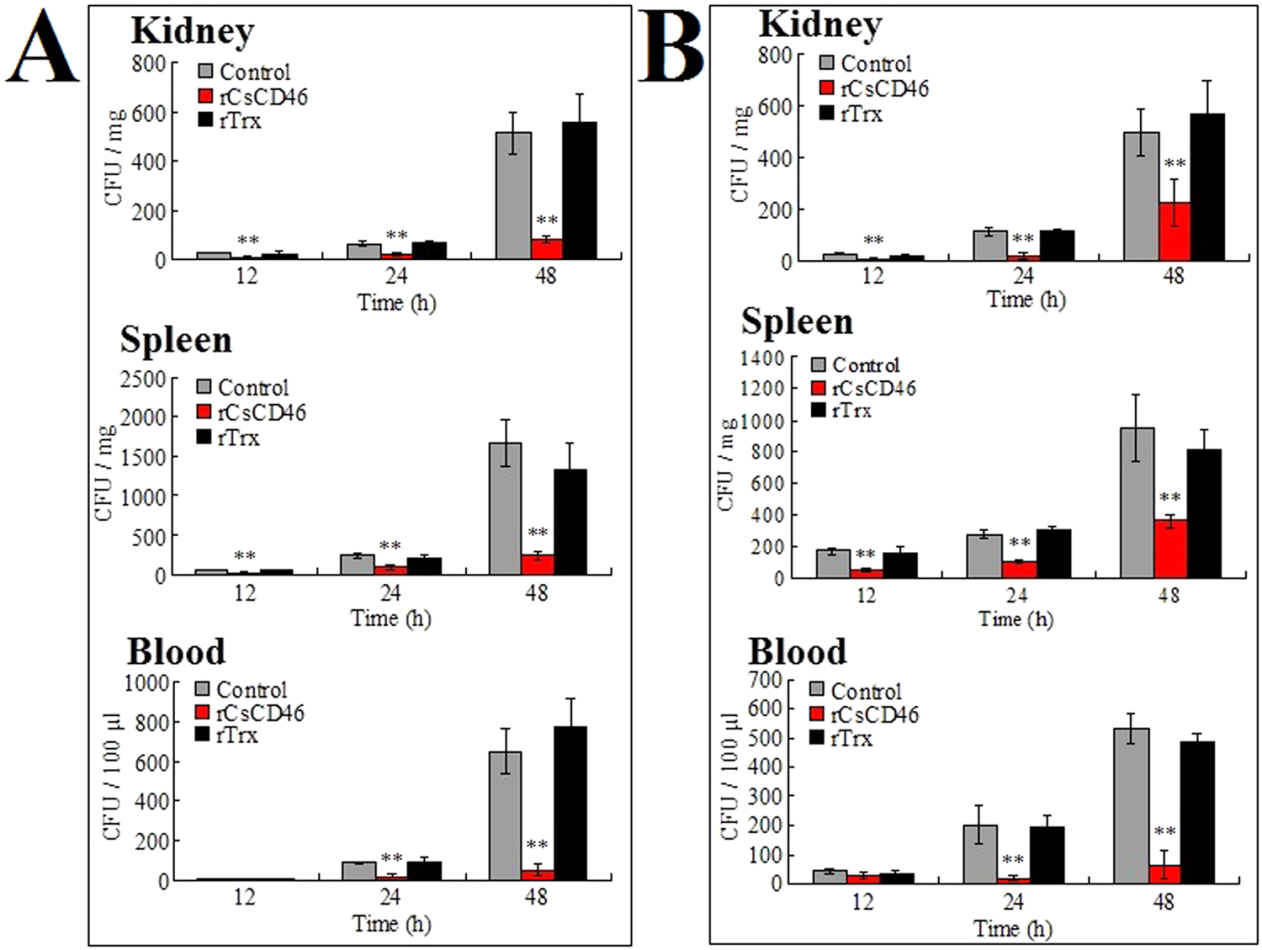
Effect of rCsCD46 on bacterial infection in tongue sole. Tongue sole were infected with *Edwardsiella tarda* (**A**) and *Pseudomonas fluorescens* (**B**) in the presence of rCsCD46, rTrx, or PBS (control), and the amounts of bacteria in kidney, spleen, and blood were determined at different times after infection. Data are the means of three independent assays and presented as means ± SEM. **P < 0.01.

To examine the *in vivo* effect of rCsCD46 on bacterial infection, tongue sole were infected with *E. tarda* and *P. fluorescens* in the presence of rCsCD46 or rTrx, and pathogen loads in the kidney, spleen, and blood of the fish were determined at 12, 24, and 48 h post-infection. The results showed that at all examined time points, the amounts of *E. tarda* recovered from all tissues in the fish infected with *E. tarda* plus rCsCD46 were significantly lower than those from the fish infected with *E. tarda* plus rTrx or *E. tarda* alone (Fig. 7A). Similarly, at 12, 24, and 48 h post-infection, the amounts of *P. fluorescens* recovered from all tissues in the fish infected with *P. fluorescens* plus rCsCD46 were significantly lower than those from the fish infected with *P. fluorescens* plus rTrx or *P. fluorescens* alone (Fig. 7B).

## Discussion

In this study, we investigated the biological role of CsCD46 in the regulation of the complement system and pathogen infection. Sequence analysis showed that CsCD46 possessed the four conserved CCP modules typical of that observed in mammalian and fish CD46. The CCP modules are known to be the sites responsible for complement regulation^25^. Consistent with the functional importance of these modules, the sequence identities/similarities between CsCD46 and human CD46 were clustered within the CCP regions. CsCD46 was similar in primary structure to most known fish CD46, however, like most fish CD46, CsCD46 does not possess the stretch of threonine residues immediately downstream of the CCP modules found in CD46 of *N. brichardi* and *P. nyererei*, suggesting that some specific features of CD46 differ among fish.

Studies in mammalian models have indicated that CD46 regulates the complement system via its ability to promote the cleavage of C3b and C4b by the serine protease factor I^12,25,26^. The CCP modules of CD46 offer binding sites for C3b, C4b, or factor I^25^. In the complement regulation process, CD46 binds to the substrate (C4b or C3b), and then factor I binds to CD46 to cleave the substrate^27–30^. In teleost, the only functional study of CD46 was carried out with the common carp CD46-like molecule Tecrem^23^. In this study, it was shown that Chinese hamster ovary cell line expressing Tecrem exhibited higher tolerance against complement damage and less deposition of C3, while an anti-Tecrem antibody enhanced the depositions of C3 and C4 on autologous erythrocytes^23^. However, the role of factor I in the action of CD46 was unclear. In our study, we found that rCsCD46 diminished the hemolytic and bactericidal activities of tongue sole serum resulting from activation of the serum complement, suggesting that rCsCD46 exerted a negative regulatory effect on complement activation. Functional CD46 in mammals is known to be a monomer, and it is possible that the rCsCD46 obtained in our study was in a monomeric form and exhibited a structure similar to that of the natural protein. We further observed that anti-rCsFI antibody significantly decreased the inhibitory effect of rCsCD46 on the complement system, most likely due to antibody blocking of natural CsFI in the serum. This conclusion was supported by the fact that serum CsFI co-immunoprecipated with rCsCD46, and that when serum was depleted of factor I by anti-rCsFI antibody absorption, the effect of rCsCD46 on serum complement was significantly reduced. These results indicated that CsCD46 was able to interact directly with CsFI to form the CsCD46–CsFI complex, which was essential for execution of the complement regulatory activity of CsCD46.

In mammals, host cells are protected from complement activation by regulatory proteins^31^. CD46 is one of such proteins that is widely expressed on most human cells, except erythrocytes and, combining with factor I, arrests propagation of the complement activation pathway by inactivation of C3b^25,32,33^. In our study, we found that CsCD46 was localized on the surface of PBL, consistent with its structural prediction, and that anti-rCsCD46 antibody significantly increased the sensitivity of PBL to serum-induced cellular destruction. These results, together with the observed complement-inhibitory effect of rCsCD46, indicate that like mammalian CD46, CsCD46 is a membrane-bound cofactor that plays a role in protecting host cells from complement attack via its capacity to control complement activation.

Previous studies have shown that some pathogens employ complement regulatory proteins to escape complement-mediated lysis^34–37^. CD46 acts as a receptor for a wide variety of viral and bacterial pathogens^38^. Measles virus and adenovirus have been reported to bind to the extracellular CCP1 and CCP2 domains of CD46, and *N. gonorrhoeae* and *N. meningitidis* bind CD46 via CCP modules and the STP region^18,39–41^. In our study, rCsCD46 was shown to interact with four Gram-negative bacterial pathogens but not with Gram-positive bacteria, possibly because of differences in the surface components between these bacterial species. Interestingly, the interaction between bacteria and rCsCD46 was unaffected by the presence of rCsFI, suggesting that rCsFI and bacteria bound to different regions of rCsCD46. *In vitro* bacterial infection analysis showed that the attachment of *E. tarda* and *P. fluorescens* to PBL was significantly reduced by the presence of anti-rCsCD46 antibody, supporting the idea that CsCD46 acts as a cellular receptor for these pathogens on PBL. In line with this idea, *in vivo* infection analysis showed that in the presence of rCsCD46, the tissue dissemination and colonization abilities of *E. tarda* and *P. fluorescens* were significantly impaired, suggesting that the interaction between the bacteria and rCsCD46 prevented binding of the bacteria to CsCD46 on host cells, thereby reducing bacterial attachment to host cells and resulting in attenuated bacterial invasion. These observations indicated that, like other pathogens that utilize CD46 for host infection, *E. tarda* and *P. fluorescens* have likely evolved the capacity to employ CD46 as a binding receptor for cellular invasion, representing the development of a virulence strategy by these pathogens during their battle with the host system.

In conclusion, we demonstrated for the first time that a teleost CD46 inhibited complement activation through recruitment of factor I, and was able to facilitate bacterial infection by serving as a cellular receptor for bacterial pathogens. These results add new insights into the function of teleost CD46 and the regulation of complement activation in fish.

## Materials and Methods

### Ethics statement

All experiments involving live animals conducted in this study were approved by the Ethics Committee of Institute of Oceanology, Chinese Academy of Sciences. All methods were carried out in accordance with the relevant guidelines, including any relevant details.

### Fish

Clinically healthy tongue sole were purchased from a commercial fish farm in Shandong Province, China. Fish were maintained at 20 °C in aerated seawater and fed daily with commercial dry pellets. Before experiment, the fish were verified to be clinically healthy by plate count as reported previously^42^. Fish were euthanized with an overdose of tricaine methanesulfonate (Sigma, St. Louis, MO, USA) before tissue collection.

### Bacterial strains and culture conditions

*E. tarda* TX1, *P. fluorescens* TSS, *V. harveyi* T4D, *V. anguillarum* C312, and *S. iniae* SF1 are bacterial pathogens isolated from diseased fish^43–45^. *E. coli* BL21 (DE3) and DH5α were purchased from TransGen (Beijing, China). *B. subtilis* was purchased from China General Microbiological Culture Collection Center (CGMCC). All strains were cultured in Luria–Bertani broth (LB) medium at 37 °C (for *E. coli* and *B. subtilis*) or 28 °C (for other bacteria).

### Sequence analysis

The amino acid sequence of CsCD46 (GenBank accession no. XP_016891345.1) was analyzed using the BLAST program at the National Center for Biotechnology Information (NCBI). Domain search was performed with the conserved domain search program of NCBI. Multiple sequence alignment was carried out with DNAMAN. O-glycosylation prediction was performed with the glycosylation site prediction tool of http://www.cbs.dtu.dk/services/NetOGlyc/.

### Preparation of cDNA

Total RNA was isolated from the tongue sole blood with EZNA Total RNA Kit (Omega Bio-tek, Doraville, GA, USA). The cDNA was synthesized with the Thermo Scientific RevertAid Reverse Transcriptase (Thermo Scientific HyClone, Beijing, China) according to manufacturer’s instructions.

### Plasmid construction

The coding sequence of CsCD46 was cloned by PCR with primers F1 (5′-ATGATGTGGAAAATCTCAGTC-3′) and R1 (5′-TCAAGAAGTTCCTGAATCACGGA-3′), which were designed based on the CD46 sequence published previously (GenBank accession no. XP_016891345.1)^46^. To construct pEtCsCD46, which expresses recombinant CsCD46 (rCsCD46) with His tags, the coding sequence of CsCD46 without the signal peptide sequence (residues 21 to 340) was amplified by PCR with primers CsCD46F (5′-GATATCATGCAAGAGTGTAATGAACCTGTG-3′, underlined sequence, EcoRV site) and CsCD46R (5′-GATATCAGAAGTTCCTGAATCACGGA-3′, underlined sequence, EcoRV site), and PCR program was 94 °C for 4 min; 5 cycles at 94 °C for 30 s, 54 °C for 30 s, 72 °C for 1 min; 25 cycles at 94 °C for 30 s, 60 °C for 30 s, 72 °C for 1 min; followed by 72 °C for 8 min. The PCR product was ligated with the T−A cloning vector T-Simple (TransGen Biotech., Beijing, China), and the recombinant plasmid was digested with EcoRV to retrieve the CsCD46-containing fragment, which was inserted into pET259^47^ at the SwaI site, resulting in pEtCsCD46. To construct pEtCsFI, which expresses His-tagged recombinant CsFI (rCsFI), CsFI without signal peptide sequence was amplified by PCR with primers CsFIF (5′-GATATCATGACAGATCCTCAAAGTCTATATT-3′, underlined sequence, EcoRV site) and CsFIR (5′-GATATCAGAATTAAACCTAGTGACTGTTG-3′, underlined sequence, EcoRV site). The PCR product was inserted into pET259 as above.

### Purification of recombinant proteins and preparation of antibody

*E. coli* BL21 (DE3) was transformed with pEtCsCD46, pEtCsFI, and pET32a (which expresses the Trx tag). rTrx was purified under the same conditions as rCsCD46 and rCsFI, so that it could be used as a control protein in subsequent experiments that analyzed the activities of rCsCD46 and rCsFI. The transformants were cultured in LB medium at 37 °C to mid-log phase, and the expression of rCsCD46, rCsFI, and rTrx were induced by adding isopropyl-β-D-thiogalactopyranoside to a final concentration of 1 mM. After growth at 16 °C for an additional 16 h, the cells were harvested by centrifugation, and recombinant proteins were purified under denaturing conditions using nickel-nitrilotriacetic acid columns (GE Healthcare, Piscataway, NJ, USA) as recommended by the manufacturer. The purified proteins were reconstituted as described previously^48^. The reconstituted proteins were treated with Triton X-114 to remove endotoxin as reported previously^49^. The proteins were dialyzed for 24 h against phosphate-buffered saline (PBS) and concentrated using PEG 20000 (Solarbio, Beijing, China). The concentrations of the purified proteins were determined using the Bradford method with bovine serum albumin as a standard. Mouse antibodies against rCsCD46, rCsFI, and rTrx were prepared as described previously^50^. The antibodies were purified using rProtein G Beads (Solarbio, Beijing, China). The specificity and titer of the serum antibodies were determined as reported previously^51^ by enzyme-linked immunosorbent assay (ELISA).

### Antibody detection of CsCD46 and CsIF in PBL and serum

Tongue sole PBL were prepared with Percoll as reported previously^52^. The proteins of ~10^9^ cells were prepared by lysing the cells with 200 µl 1% Triton X-100. The proteins were resolved by sodium dodecyl sulfate-polyacrylamide gel electrophoresis (SDS-PAGE) and transferred to a nitrocellulose membrane. Western blot was performed as reported previously^49^ with anti-rCsCD46 antibody (1 µg/ml). To detect CsFI in serum, blood was drawn from the caudal vein of tongue sole and placed on ice immediately. The blood was allowed for clotting for 2 h. Serum was then collected by centrifugation at 4000 rpm for 10 min. For Western blot analysis, the serum was concentrated approximately 20-fold and subjected to SDS-PAGE; the proteins were then transferred to a nitrocellulose membrane, and Western blot was performed as above with anti-rCsFI antibody. Western blot examining binding of anti-rCsFI and anti-rCsCD46 antibodies to rCsFI and rCsCD46, respectively, was performed as above. The results showed that anti-rCsCD46 and anti-rCsFI antibodies detected CsCD46 and CsFI, respectively, in PBL and serum (Fig. S3). The molecular mass of the natural CsCD46 appeared to be larger than the theoretical molecular mass, which is probably due to glycosylation of the protein.

### Complement activity assay

Complement activity was determined as reported previously^53^. To examine the effect of rCsCD46 on complement activation, tongue sole serum was diluted serially in Hank′s Balanced Salt Solution (HBSS) (Solarbio, Beijing, China). Inactivated serum was prepared by heating at 56 °C for 30 min and serum dilutions were then added with or without rCsCD46 or rTrx to a final concentration of 40 µg/ml. The mixtures were then incubated at 22 °C for 1 h. To measure hemolytic activity, rabbit red blood cells (Guangzhou Future Technology Co., Ltd, Guangzhou, China) were washed and resuspended in HBSS, and 10 µl of suspension was then added to 50 µl of each of the serum mixtures or heat-inactivated serum in a 96-well culture plate, followed by incubation at 22 °C for 30 min. After incubation, the supernatant was collected by centrifugation and the absorbance at 450 nm was determined. For easy of comparison, the hemolytic activity of the control sample at 1/8 serum dilution was defined as 1. For bactericidal activity assay, the above serum mixtures were either untreated or heated at 56 °C for 30 min. *E. coli* DH5α was cultured in LB medium to an optical density (OD)_600_ of 0.8; the cells were washed and resuspended to 2 × 10^6^ colony-forming units (CFU)/ml in HBSS. The bacterial suspension was combined with an equal volume of heated or unheated serum mixture. After incubation at 22 °C for 1 h, the sample was serially diluted and plated in triplicate in LB agar plates. The plates were incubated at 28 °C for 24 h, and the colonies that appeared on the plates were enumerated. The genetic nature of the colonies was verified by PCR. For easy of comparison, the bactericidal activity of the control sample at 1/8 serum dilution was defined as 1.

### Co-immunoprecipitation

Interaction between rCsCD46 and CsFI was determined by co-immunoprecipitation using an Immunoprecipitation Protein A/G Plus Agarose Kit (Sangon Biotech, Shanghai, China) according to the manufacturer’s instructions. Tongue sole serum containing rCsCD46 (20 µg/ml, 40 µg/ml, or 80 µg/ml) or rTrx (80 µg/ml) was mixed with *E. coli* (10^6^ CFU/ml) and incubated at 22 °C for 1 h. The mixture was passed through a 0.22-µm microfilter to remove bacterial cells. The filtered serum was treated with anti-His antibody (1 µg/ml) at 4 °C for overnight. After treatment, the mixture was transferred to a spin column bound with protein-A/G and incubated at 4 °C for 2 h. After incubation, the spin column was centrifuged and washed three times. Fifty microliters loading buffer was added to the column, and the column was treated at 95 °C for 5 min. The immunoprecipitated complex was collected by centrifugation and concentrated 20-fold with PEG 20000. The immunoprecipitated complex and rCsFI protein (positive control) were resolved by SDS-PAGE and transferred to a nitrocellulose membrane (Amersham, Cambridge, UK). Western blot was performed as above with anti-rCsFI antibody (1 µg/ml). The membrane of Western blot was washed with PBS and detected with the HRP-DAB kit (Tiangen, Beijing, China). The color reaction was stopped after 10 min by several washes with water, and the membrane was air dried.

Interaction between rCsCD46 and rCsFI was determined by co-immunoprecipitation as above. rCsCD46 (80 µg/ml) was mixed with rCsFI (80 µg/ml) and incubated at 22 °C for 1 h. The mixture was treated with anti-rCsCD46 antibody (1 µg/ml) at 4 °C for overnight. Co-immunoprecipitation and Western blot were performed as above.

### Involvement of CsFI in rCsCD46-mediated effect on complement activation

To examine the effect of anti-rCsFI antibody on rCsCD46-mediated inhibition of complement activation, tongue sole serum was diluted serially in HBSS. Anti-rCsFI antibody or preimmune antibody (2 µg/ml) was mixed with the serum dilutions in the presence or absence of 40 µg/ml rCsCD46 or rTrx. The control serum was mixed with the same volume of HBSS. The mixtures were incubated at 22 °C for 1 h, and hemolysis activity was measured as above. To examine the effect of CsFI depletion on rCsCD46-mediated inhibition of complement activation, tongue sole serum was treated with anti-rCsFI antibody (1 µg/ml) or anti-rTrx antibody (1 µg/ml) at 4 °C for overnight. After incubation, the mixture was transferred to the spin column bound with protein-A/G and incubated at 4 °C for 2 h. The spin column was then centrifuged, and the serum was collected. The serum was diluted serially in HBSS. rCsCD46 was mixed with anti-rCsFI antibody-treated serum, anti-rTrx antibody-treated serum (control serum), or normal serum, and the mixture was determined for hemolytic activity as above.

### Fluorescence microscopy

Tongue sole PBL were prepared with Percoll as above. Detecting of CsCD46 on PBL by fluorescence microscopy was performed as reported previously^54^. Briefly, PBL were resuspended in PBS to 10^7^ cells/ml. Anti-rCsCD46 antibody or anti-rTrx antibody (2 µg/ml) was added to PBL suspension. The cells were incubated at 22 °C for 2 h and then centrifuged at 300 *g* for 10 min. The cells were collected, washed three times with PBS, and resuspended in PBS. Fluorescein isothiocyanate (FITC)-labeled goat anti-mouse IgG (Bioss, Beijing, China) (1/1000 dilution) was added to the cells, followed by incubation at 22 °C for 2 h in the dark. The cells were centrifuged and washed as above. The cells were resuspended in PBS and observed with a fluorescence microscope (Nikon E800, Japan).

### Flow cytometry analysis of cell damage

PBL prepared above were resuspended in L-15 medium (Thermo Scientific HyClone, Beijing, China) to 1 × 10^7^ cells/ml. Anti-rCsCD46 antibody, anti-rTrx antibody, anti-rCsTLR2 antibody^55^, preimmune antibody, all at 2 µg/ml, or PBS was added to PBL. After incubation at 22 °C for 1 h, the cells were centrifuged at 300 *g* for 10 min. The cells were washed three times with PBS and resuspended in serum, followed by incubation at 22 °C for 1 h. The control cells were untreated with serum or antibody. To measure cellular damage, PBL were treated with annexin V and propidium iodide (PI) (Majorbio Biotech, Shanghai, China) for 15 min in the dark according to the manufacturer’s instructions. The cells were then subjected to flow cytometry using a FACSort Flow Cytometer (BD Biosciences, USA). Data analysis was performed using FlowJo software 7.6.1 (Tree Star Inc, Ashland, OR, USA). To examine the effect of factor I on CsCD46-induced protection of PBL against complement damage, anti-rCsFI antibody or anti-rTrx antibody (2 µg/ml) was mixed with serum and incubated at 22 °C for 1 h. Treatment of PBL with the serum and measure of cellular damage were performed as above.

### Binding of rCsCD46 to bacteria

Bacteria were cultured in LB medium to OD_600_ 0.8 and resuspended in coating buffer (15 mM Na_2_CO_3_, 35 mM NaHCO_3_, pH 9.6) to 10^8^ CFU/ml. Bacteria–protein interaction was determined by ELISA as reported previously^56^. Briefly, the bacterial suspension was added to 96-well ELISA plates and incubated at 4 °C for overnight. rCsCD46 was heated at 100 °C for 10 min to prepare inactivated rCsCD46. After blocking with 5% skim milk and washing with PBST (PBS containing 0.1% Tween- 20), different concentrations (2.5 µg/ml, 5 µg/ml, 10 µg/ml, 20 µg/ml, 40 µg/ml, and 80 µg/ml) of rCsCD46, heat inactivated rCsCD46, rTrx, or PBS were added to the plates, and the plates were incubated at 22 °C for 2 h. The plates were then washed with PBST, and mouse anti-His antibody (Tiangen, Beijing, China) and horseradish peroxidase (HRP)-conjugated goat anti-mouse IgG (Bioss, Beijing, China) were added to the plates sequentially as reported previously^56^. The TMB Kit (Tiangen, Beijing, China) was used to observe color development. The plates were read at 450 nm using a Precision microplate reader (Molecular Devices, Toronto, Canada). Positive readings were defined as values at least twice that of the control. The assay was performed three times and the results were expressed as the binding index, defined as follows: *A*_450_ of protein/*A*_450_ of PBS.

To examine the effect of rCsCD46 on bacterial survival, *E. tarda* and *P. fluorescens* (10^4^ CFU) were incubated with 40 µg/ml rCsCD46, rTrx, or PBS for 4 h at 22 °C. The cells were then diluted, and the dilutions were plated in triplicate on LB agar plates. The plates were incubated at 28 °C for 24 h, and the bacterial numbers were counted.

To determine the effect of rCsFI on the interaction between rCsCD46 and bacteria, *P. fluorescens* suspension was prepared as above and added to a 96-well ELISA plate. The plate was incubated at 4 °C, blocked with skim milk, and washed with PBST as above. PBS (control), or rCsCD46 plus rCsFI, rCsCD46 plus rTrx, rCsCD46, rCsFI, or rTrx (all proteins being at 40 µg/ml) was added into the plate. The plate was incubated at 22 °C for 2 h and washed as above. ELISA was performed as above.

### Effect of anti-rCsCD46 antibody on bacterial attachment to PBL

Anti-rCsCD46 antibody (1 µg/ml), anti-rTrx antibody (1 µg/ml), preimmune antibody (1 µg/ml), or PBS was added to PBL. The cells were incubated at 22 °C for 1 h. *E. tarda* and *P. fluorescens* were cultured in LB medium to an OD_600_ of 0.8 and resuspended in PBS to 1 × 10^6^ CFU/ml and 1 × 10^7^ CFU/ml, respectively. One hundred microliters of bacterial suspension was added to each well of PBL (~10^5^ cells/well). The plate was incubated at 22 °C. At 1, 2, and 4 h post-incubation, the cells were washed three times with PBS to remove uninfected bacteria. The cells were lysed by adding 100 µl of 1% Triton X-100; the lysate was diluted in LB medium and plated on LB agar plates. The plates were incubated at 28 °C for 24 h, and the colonies that emerged on the plates were counted. The genetic nature of the colonies was verified by PCR with primers reported previously^57^.

### Effect of rCsCD46 on bacterial infection in fish

*E. tarda* and *P. fluorescens* were cultured as above and resuspended in PBS to 2 × 10^6^ CFU/ml and 2 × 10^7^ CFU/ml, respectively. rCsCD46 or rTrx was added to the cells to the final concentration of 40 µg/ml. The control cells were added with PBS. Tongue soles were divided randomly into three groups and injected intramuscularly with 50 μl suspensions of bacteria, bacteria plus rCsCD46, or bacteria plus rTrx. At 12 h, 24 h, and 48 h post-infection, kidney, spleen, and blood were collected from the fish (five at each time point). The tissues were homogenized in PBS. The homogenates were serial diluted and plated in triplicate on LB agar plates. After incubation at 28 °C for 48 h, the colonies that appeared on the plates were enumerated. The genetic identification of these colonies was verified by PCR as above.

### Statistical analysis

All experiments were repeated three times. Statistical analyses were carried out with SPSS 17.0 software (SPSS Inc., Chicago, IL, USA). Data were analyzed with analysis of variance (ANOVA), and statistical significance was defined as *P* < 0.05. In Fig. 3A and B, statistical significance represented by different letters was defined as *P* < 0.05.

## Electronic supplementary material

Supplementary information
