# Supplementary material for: A teleost CD46 is involved in the regulation of complement activation and pathogen infection
Source: Sci Rep. 2017 Nov 3;7:15028. doi: 10.1038/s41598-017-15124-y (PMC5670209; doi:10.1038/s41598-017-15124-y)

**A teleost CD46 is involved in the regulation of complement activation and pathogen infection**

Mo-fei Li^1,2^, Zhi-hai Sui^1,3^, Li Sun^1,2^*

*^1^Key Laboratory of Experimental Marine Biology, Institute of Oceanology, Chinese Academy of Sciences, Qingdao, China*

*^2^Laboratory for Marine Biology and Biotechnology, Qingdao National Laboratory for Marine Science and Technology, Qingdao, China*

*^3^University of Chinese Academy of Sciences, Beijing, China*

*To whom correspondence should be addressed

Mailing address: Li Sun

Institute of Oceanology

Chinese Academy of Sciences

7 Nanhai Road

Qingdao 266071, China

Phone: 86-532-82898829

Email: lsun@qdio.ac.cn

**Supplemental data**

**Figure S1.** SDS-PAGE analysis of purified rCsCD46, rCsFI, and rTrx. Purified rCsCD46, rCsFI, and rTrx (lanes 2, 3, and 4 respectively) were analyzed by SDS-PAGE and viewed after staining with Coomassie brilliant blue R-250. Lane 1, protein markers.

**
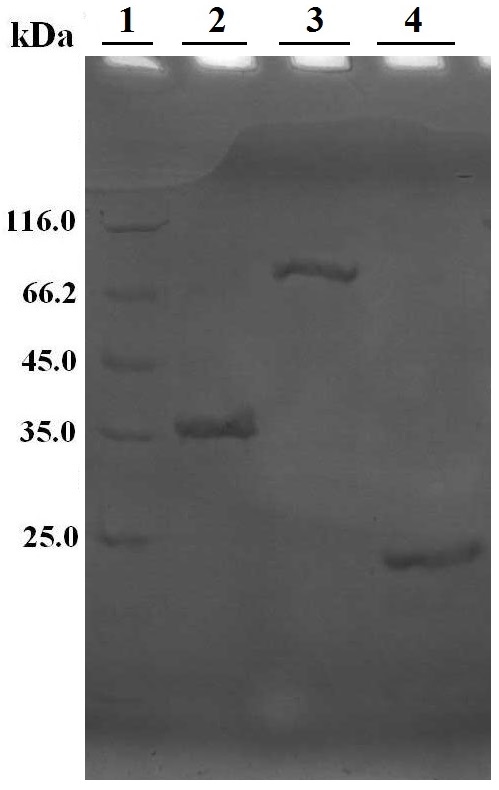
**

**Figure S2.** The effect of CsFI on CsCD46-induced protection of peripheral blood leukocytes (PBL). PBL were incubated with anti-CsFI antibody-treated serum or anti-rTrx antibody-treated serum for 1 h; the control cells were untreated with serum or antibody. Cellular damage was then determined by Annexin V-PI assay.

**
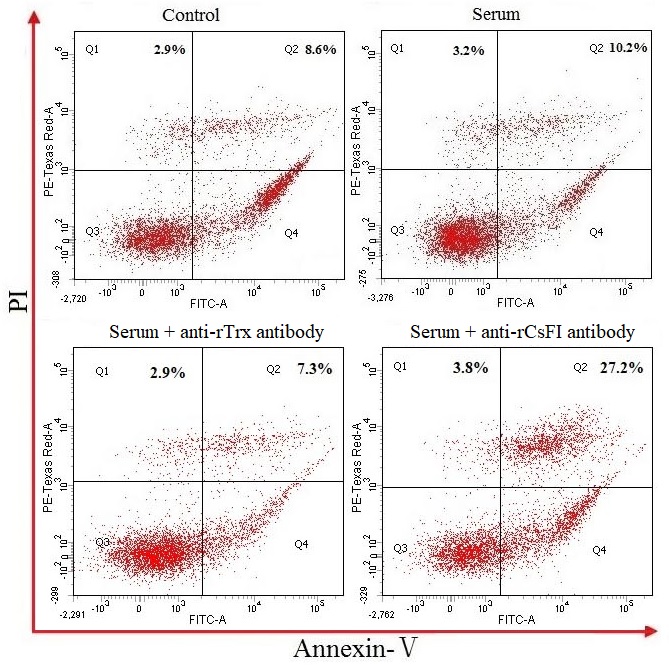
**

**Figure S3.** Antibody detection of CsCD46 and CsFI in tongue sole peripheral blood leukocytes (PBL) and serum, respectively. (A) PBL proteins (lanes 2) were subjected to immunoblot with rCsCD46 antibody. (B) Serum (lanes 2) was subjected to immunoblot with rCsFI antibody. (C) rCsCD46 (lanes 2) was subjected to immunoblot with rCsCD46 antibody. (D) rCsFI (lanes 2) was subjected to immunoblot with rCsFI antibody. Lane 1 of all panels, protein markers.


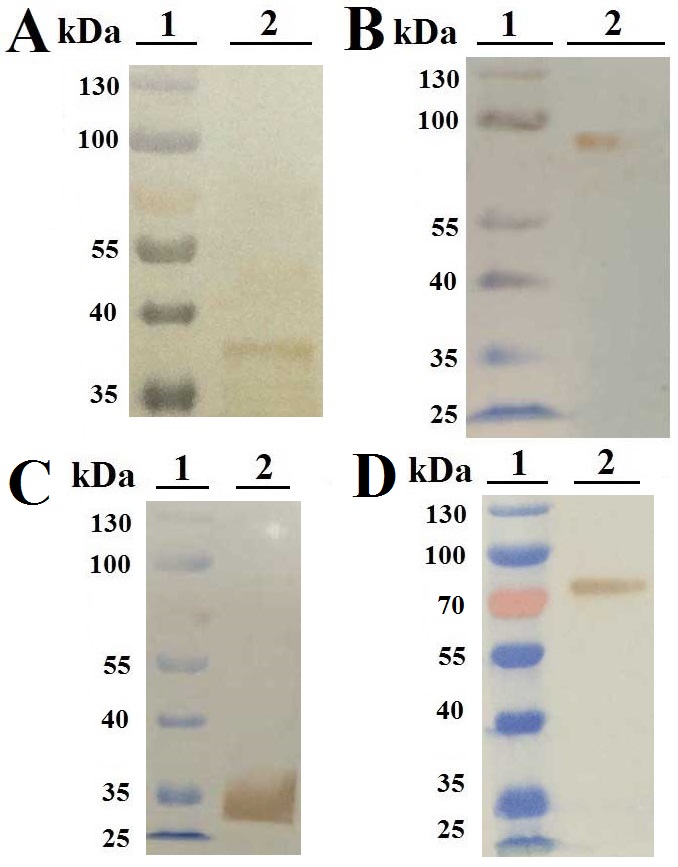

Supplement: Supplementary file 1 — Supplementary information [file 41598_2017_15124_MOESM1_ESM.doc]
